# Comparison of Different Clinical Chemotherapeutical Agents’ Toxicity and Cell Response on Mesenchymal Stem Cells and Cancer Cells

**DOI:** 10.3390/cells11192942

**Published:** 2022-09-20

**Authors:** Flóra Vajda, Áron Szepesi, György Várady, Judit Sessler, Dániel Kiss, Zsuzsa Erdei, Kornélia Szebényi, Katalin Német, Gergely Szakács, András Füredi

**Affiliations:** 1 Institute of Enzymology, Research Centre for Natural Sciences, 1117 Budapest, Hungary; 2 Doctoral School of Molecular Medicine, Semmelweis University, 1089 Budapest, Hungary; 3 Creative Cell Kft., 1119 Budapest, Hungary; 4 Software Engineering Institute, John von Neumann Faculty of Informatics, Óbuda University, 1034 Budapest, Hungary; 5 National Laboratory for Drug Research and Development, 1117 Budapest, Hungary

**Keywords:** tumor microenvironment, mesenchymal stem cells, cancer cells, chemotherapeutics, drug tolerance

## Abstract

Mesenchymal stem cells (MSCs) or fibroblasts are one of the most abundant cell types in the tumor microenvironment (TME) exerting various anti- and pro-apoptotic effects during tumorigenesis, invasion, and drug treatment. Despite the recently discovered importance of MSCs in tumor progression and therapy, the response of these cells to chemotherapeutics compared to cancer cells is rarely investigated. A widely accepted view is that these naive MSCs have higher drug tolerance than cancer cells due to a significantly lower proliferation rate. Here, we examine the differences and similarities in the sensitivity of MSCs and cancer cells to nine diverse chemotherapy agents and show that, although MSCs have a slower cell cycle, these cells are still sensitive to various drugs. Surprisingly, MSCs showed similar sensitivity to a panel of compounds, however, suffered fewer DNA double-stranded breaks, did not enter into a senescent state, and was virtually incapable of apoptosis. Our results suggest that MSCs and cancer cells have different cell fates after drug treatment, and this could influence therapy outcome. These findings could help design drug combinations targeting both MSCs and cancer cells in the TME.

## 1. Introduction

According to the WHO statistics, 9.9 million people died from cancer in 2020 [1]. Chemotherapy is the most often used treatment strategy against advanced and disseminated tumors, as an estimated 60% of all cancer patients receive chemotherapeutics [2]. To control tumor growth, conventional chemotherapy agents target rapidly dividing cancer cells, exploiting vulnerabilities linked to increased DNA synthesis and enhanced use of cell division machinery. Unfortunately, the effects of chemotherapy are often short-lived, as cancer cells develop various mechanisms to cope with continuous drug exposure.

Recently, the tumor microenvironment (TME) attracted increasing attention as a possibly important contributor to drug resistance. TME consists of immune cells, vascular endothelial cells, and mesenchymal stem cells (MSCs) or fibroblasts, which can have a critical role in tumor progression, metastasis, and drug resistance. MSCs are multipotent cells, with self-renewable capacity, immunosuppressive effect, tissue regeneration, and high migration trait. These cells can be isolated from different sources, such as adipose tissue, bone-marrow, umbilical cord, dental pulp, or dermal tissue. MSCs have the potential to migrate to the primary tumor site, mostly from the local adipose tissue and the distant bone marrow niche due to endocrine and paracrine signals (bFGF, HDGF, MCP-2, UPA) secreted by the cancer cells [3]. These MSCs can form a major subpopulation in the tumor stroma, for example, in breast- and pancreatic carcinoma, the MSCs ratio can be nearly 80% of the whole tumor mass [4]. Because of the complexity and cellular heterogeneity of the TME, there is an intensive crosstalk between the different cell types [5].

MSCs, as one of the most abundant cell types in TME [6], are known to affect neighboring cells directly or indirectly. Through releasing a complete arsenal of different cytokines, chemokines, and matrix metalloproteinases (MMP1, MMP2), MSCs can reorganize the extracellular matrix or by secreting interleukins (e.g., IL-6) can alter the behavior and the gene expression profile of other cells. These multipotent cells can have a direct and dynamic interaction with cancer cells via membrane- and microvesicules/exosomes exchange, cell fusion, or by transferring different proteins and mRNAs [7].

MSCs can have a somewhat controversial dual role during tumor progression: these cells can exert either pro-tumorigenic or antitumoral effects depending on the phase of the process. Their primary anti-cancer activity—expressing antiangiogenic agents (PDGF), cytotoxic agents (TRAIL, INF-γ), anti-proliferation factors (Wnt, BMP, PTEN), and immunomodulators targeting NK-cells, B-cells, and T-cells. Later, pro-tumorigenic activity will prevail by secreting survival factors (bFGF, HGF, IGF1), pro-angiogenic agents (VEGF, TGFB), immunosuppressive cytokines (IDO, TGFB, IL8, IL10), and transform to cancer-associated fibroblast (CAF) [7].

According to the widely accepted view, chemotherapeutics causes less damage to healthy/non-proliferating/non-tumor cells, targeting only the highly proliferative cancer cells. Our initial aim was to test nine, structurally and mechanistically different, clinically applied anticancer agents in vitro both on MSCs and cancer cells to identify cellular mechanisms helping healthy cells survive under various chemotherapy treatment/conditions. Surprisingly, in our drug sensitivity screening, we found that, while there are some compounds that kill cancer cells significantly more efficiently, 5 out of 9 molecules were equally toxic to both cancer cells and MSCs. These striking results challenge the basics of conventional chemotherapy, shed light on the complex interplay between cancer cells and the TME, and could lead to novel approaches in therapy design.

Here, we compare the response of cancer cells and MSCs isolated from healthy donors to a panel of chemotherapeutic agents, investigate the effect of these treatments on DNA damage, reactive oxygen species production, apoptosis, and cellular senescence, while we try to dissect the differences in the reaction to these treatments.

## 2. Materials and Methods

### 2.1. Cell Culture

To compare drug sensitivity, a panel of six MSCs and three cancer cell lines were used.

Three human primary adipose tissue-derived mesenchymal stem cells, Ad-MSC 1-3, an immortalized Ad-MSC 1 (iAd-MSC 1), originating from the Ad-MSC 1 primary cell line with lentiviral mediated overexpression of polycomb complex protein Bmi-1, and human telomerase reverse transcriptase (hTERT) according to Tátrai et al. (2012) were used [8]. Bone-marrow-derived mesenchymal stem cell (BM-MSC) was isolated and characterized by our group, according to Szepesi et al. (2016). Detailed characterization of the BM-MSC cell line is shown in Supplementary Figure S1. Human foreskin fibroblast (HFF), human skin epidermoid carcinoma (A431), and human uterine sarcoma (MES-SA) cells were purchased from ATCC, and the breast adenocarcinoma (MCF-7) cell line was obtained from the National Cancer Institute’s Developmental Therapeutics Program. Cells were cultured in Dulbecco’s modified Eagle’s medium (DMEM-F12, Gibco, Waltham, Massachusetts) supplemented with 10% fetal bovine serum (FBS, Gibco), 1% L-glutamine (Gibco), 0.1% gentamicin (Gibco, 50 mg/mL), and 16 ng/mL fibroblast growth factor 2 (Peprotech, London, UK). Cells were kept at 37 °C and 5% CO_2_. At approximately 80% confluency, cells were trypsinized (0.1% for MSCs and 0.2% for cancer cells) for between 2 and 10 min. Followed by centrifugation (1500 rpm/5 min), resuspension, and replating for expansion. The medium was renewed every 72 h on each cell line and MSCs were used up to passage 25. GFP or mCherry fluorescent protein expressing sublines were previously established with lentiviral transduction by our group: Ad-MSCs-GFP, immortalized Ad-MSC-GFP 1, A431-mCh, MES-SA-mCh, and MCF-7-GFP according to Tátrai et al. [9]. Drug sensitivity of Ad-MSC-GFP 3 and A431-mCherry is similar to their parental cell lines (Supplementary Figure S2).

### 2.2. Drugs and Chemicals

Chemotherapeutic agents were purchased from different sources: cisplatin (Vnr461201, Accord Healthcare, UK), methotrexate (M9929, Sigma Aldrich, St. Louis, MO, USA), irinotecan hydrochloride (AB252173, abcr, Karlshure, Germany), doxorubicin hydrochloride (25316-40-9, Sigma Aldrich, United States), vinblastine (HY-13780, MedChemExpress, Monmouth Junction, NJ, USA), TPEN (P4413, Tocris, Bristol, UK), nutlin-3 (3984, Tocris, UK), mitoxantrone (M2305000, Sigma Aldrich, UK), and bendamustine was a kind gift from Servier.

### 2.3. Cytotoxicity Assay and Cell Viability Assay

Cells were seeded in a 96-well tissue culture plate in 100 µL of medium. Ad-MSC-GFP 1, iAd-MSC-GFP 1, Ad-MSC-GFP 2, Ad-MSC-GFP 3, BM-MSC, and HFF were seeded at 5 × 10^3^/100 µL/well density and A431-mCh, MES-SA-mCh, and MCF-7-GFP at 4 × 10^3^ cells/100 µL/well concentration in DMEM-F12 media. Different initial cell numbers were used due to different proliferation rates of different cell types. Cells were allowed to attach overnight at 37 °C and the day after were treated with the drugs using the indicated concentrations in 100 µL. Cell viability was measured by using PrestoBlue™ Cell Viability Reagent (A13232, Invitrogen, Waltham, MA, USA). To address metabolic differences between cancer cells and MSCs, the assay was optimized for each cell line, cells were incubated with 5 or 12.5% PrestoBlue™ Cell Viability Reagent diluted in PBS at 37 °C/5% CO_2_ for 1–1.5 h 5 days after the treatment. The fluorescence signal was measured by an EnSpire (Perkin Elmer, Waltham, MA, USA) fluorometer at 555 nm (excitation)/585 nm (emission) wavelength.

### 2.4. Dose–Response Curves and IC_50_ Calculation

After the incubation period, the relative fluorescence data was measured. The data was normalized with the fluorescence of blank samples (cell culture media). A sigmoidal dose–response curve was fitted to the normalized measurement data and the logarithm of the concentration using the least square method. An inhibitory concentration of 50% (IC_50_ and log10) was interpolated from the fitted graph. In the case of co-culture, we experienced that at low concentrations the data did not fit the sigmoidal curve. In these cases, IC_50_ was calculated from the line connecting the measurement points. For repeated measurements, average and standard deviation was calculated based on the logarithm of the IC_50_ values.

### 2.5. Apoptosis Assay

Apoptosis detection was based on the Annexin-V molecule binding to phosphatidylserine membrane molecules. Two cell lines were used to study apoptosis: Ad-MSC 3 cells were plated in a 5 × 10^4^ cells/well concentration, while 4 × 10^4^/well A431-mCh were seeded into each well in a 12-well tissue culture plate. Cells were incubated overnight, and treated with cisplatin, doxorubicin, and nutlin-3 at the indicated concentrations in 1 mL of culture media. At day 5, cells were trypsinized and centrifuged, while the supernatant was also collected and combined with the detached cells to include all already dead cells. Cells were washed twice with 500 µL of 1% BSA-PBS (bovine serum albumin dissolved in PBS, A8022, Sigma-Aldrich, St. Louis, MO, USA) solution and centrifuged at 400× *G* for 4 min. Cells were resuspended in 100 µL of 1× Annexin Binding Buffer (422201, Biolegend, San Diego, CA, USA), and 3 µL of Annexin V-Pacific Blue (A35122, Biolegend, San Diego, CA, USA) and 1 µL of TO-PRO-3 (R37170, Invitrogen, Waltham, MA, USA) were added to each sample. After 15 min of incubation, labeled and non-labeled samples were diluted 5-fold with 1× Annexin Binding Buffer and analyzed by Attune™ NxT flow cytometer.

### 2.6. Cell Proliferation Analysis

Cell division was analyzed by the fluorescent CytoTell Green dye (AAT Bioquest, Sunnyvale, CA, USA) according to the manufacturer’s guidelines. Briefly, 3 × 10^5^ cells were centrifuged and stained with 2 µL of CytoTell dye diluted in 1 mL of serum-free DMEM and incubated at 37 °C for 30 min. After centrifugation, half of the cells (1.5 × 10^5^) were plated into T25 cell culture flasks in 5 mL of media and analyzed at day 5. Day 1 samples were co-stained with Zombie Violet dye (423113, Biolegend) to exclude dead cells and analyzed by Attune™ NxT flow cytometry.

### 2.7. Immunocytochemistry

Ad-MSC-GFP 3 cells were seeded into glass-bottomed chamber slides (ibidi, Germany, Grafelfing, Germany) at 1 × 10^4^/well and A431-mCh at 1.6 × 10^4^/well density. The day after, cells were treated with 2 µM of cisplatin, 0.1 µM of doxorubicin, and 30 µM of nutlin-3 diluted in 200 µL of DMEM-F12. Cells in all chambers were fixed with 4% PFA for 15 min and washed twice with PBS. Blocking solution (0.5% BSA-PBS, 0.1% TritonX-100, 5% goat serum, and 1% fish gelatin) was used for 1 h. γ-H2A.X primary antibody (MA1-2022, Invitrogen, United States) was diluted in blocking solution at 1:500 concentration and incubated overnight at 4 °C. Afterwards, the cells were washed with PBS twice and goat anti-mouse A633 secondary antibody (A21052, abcam, Cambridge, UK) was diluted at 1:250 concentration in blocking solution. Nuclei was stained with a Hoechst-33342 (23491-52-3, Dojindo, Kumamoto, Japan) at 1:1000 concentration. Cells were observed with a Zeiss LSM-710 confocal microscopy at 40× magnification with the same settings for all samples.

### 2.8. γ-H2AX Image Analysis

To quantify DNA damage based on immunofluorescence against γ-H2AX, a semi-automated image analysis pipeline was set up using CellProfiler v4.1.3 [10]. First, images showing DAPI-stained nuclei were pre-processed by intensity normalization and Gaussian filtering (ϭ = 20 px) to suppress noise and remove artifacts caused by debris. Nuclei candidates were identified by threshold-based segmentation; false candidates were removed from the analysis manually. Using the remaining nuclei regions as masks, pixel intensities were measured on the γ-H2AX channel, thus, excluding non-nuclei signals.

### 2.9. ROS Induction

Ad-MSC 3 and A431 cells were seeded in a 96-well tissue culture plate and incubated for 24 h as described above. Cells were washed twice with HBSS and incubated in 100 µL of DCFH-DA dye (R252-10, Dojindo) according to the manufacturer’s instructions. Cells were incubated for 30 min at 37 °C and washed twice with HBSS. Cells were treated with 200 µL of cell culture medium with appropriate concentrations of H_2_O_2_, cisplatin, doxorubicin, and nutlin-3 for 1 h. Supernatant was removed, and the cells were washed with HBSS before imaging with the JuLI^TM^ Stage Cell History Recorder (NanoEnTek).

### 2.10. Senescence Staining

Ad-MSC 3 and A431 cells were seeded in a 96-well tissue culture plate and treated with cisplatin, doxorubicin, or nutlin-3. At day 5, media was removed and replaced with 200 µL of completed DMEM-F12 for 7 days. The Senescence β-Galactosidase Staining Kit (9860S, Cell Signaling Technology, Danvers, MA, USA) was used to detect senescent cells. Cells were fixed with fixing solution, washed with PBS, stained with X-gal solution overnight at 37 °C without CO_2_, and coated with 70% glycerol.

### 2.11. Live Cell Imaging

JuLI™ Stage Cell History Recorder was used to monitor a five-day cytotoxicity assay on Ad-MSC-GFP 3 and A431-mCh cell lines. Cells were seeded and treated with cisplatin, doxorubicin, and nutlin-3 as described above. Images were taken every 105 min for 120 h at 10× objective magnification. Live cell microscopy studies were evaluated using the “Growth curve” function of the JuLI STAT image analysis software (v2.0.1.0).

Brightfield images were taken with the Olympus IX51 microscope. The scale bar is the same in every image.

### 2.12. Statistical Analysis

To compare the mean IC_50_ values of six mesenchymal stem cell lines (Group-1) and three cancer cell lines (Group-2), first an F-test was performed to check equal variances between the groups, then two-sample unpaired *t*-tests were performed to calculate significance using Microsoft^®^ Excel^®^ 2016 (16.0.4266.1001).

## 3. Results

### 3.1. MSCs and Cancer Cells Show Different Response Patterns to a Panel of Chemotherapeutic Agents

**Figure 1:**
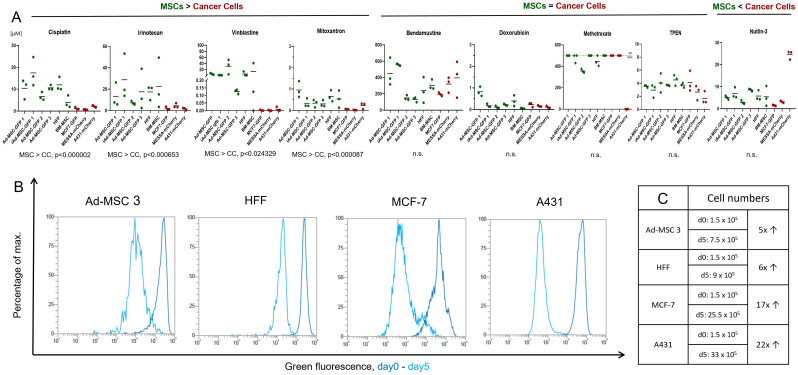
Investigation of drug sensitivity and proliferation rate of MSCs and cancer cells. (**A**) IC_50_ values (µM) of nine different compounds measured in 6 MSCs and 3 cancer cell lines. Results expressed as a mean of at least 3 repeated experiments with standard deviation. (**B**) Ad-MSC 3, HFF, MCF-7, and A431 cell lines’ proliferation rates were compared using the CytoTell Green dye. Dark blue represents the fluorescent signal at day 0 and light blue at day 5. Larger gap between the two curves means greater proliferation. (**C**) Cell numbers were counted at days 0 and 5.

To assess the drug sensitivity of MSCs and cancer cells we determined the IC_50_ value of nine different compounds on six different human MSCs and three tumor cell lines (Figure 1A). Four of the nine compounds, cisplatin, an interstrand crosslinking agent, irinotecan, a topoisomerase I inhibitor, mitoxantrone, a topoisomerase II inhibitor, and the anti-microtubule drug vinblastine required significantly higher concentrations to kill MSCs than cancer cells, showing increased selectivity towards malignant cells. Moreover, MSCs tolerated vinblastine treatment so strikingly well that the IC_50_ values were 1000-fold higher than that measured for cancer cells (‘MSCs > Cancer Cells’ group). Nutlin-3 was selected based on its specific mechanism to target cells expressing wild-type (wt) p53. Due to almost every cell line in this study harboring the wt p53 gene, except A431, which is a mutant, nutlin-3 was equally toxic among different cell lines (‘MSCs < Cancer Cells’ group). A431 showed between 3- and 10-fold resistance to the treatment. The third group of compounds were characterized by similar toxicity to both MSCs and cancer cells. Doxorubicin (topoisomerase II inhibitor), bendamustine (intrastrand crosslinking agent), methotrexate (antimetabolite), and TPEN (ion chelator) possess IC_50_ values in the same concentration range for all cell lines (‘MSCs = Cancer Cells’ group), however, A431 cells proved to have extreme sensitivity to methotrexate. Initially, an additional DNA methylating prodrug, temozolomide, mostly used to treat brain tumors, was included in the screening, but it was not toxic even at 1000 µM, suggesting neither of these cell lines are sensitive to DNA methylation.

The results obtained in the ‘MSCs = Cancer Cells’ compound group are even more surprising considering the differences in proliferation rate between MSCs and cancer cells. Proliferation was assessed at day 0 and day 5 in 4 cell lines, 2 MSCs, and 2 cancer cells (Ad-MSC 3, HFF, MCF-7, and A431). CytoTell fluorescence gradually decreases by every cell division due to the dilution of the dye, providing a measure for the division rate. During the 5-day observation period, the fluorescence signal of cancer cells reduced by 100-fold compared to an approximately 10-fold change in MSCs, suggesting a significantly lower proliferation potential in non-cancerous cells (Figure 1B). Monitoring the actual cell count in the same period confirmed the results of the fluorescence labeling: cell number in cancer cell cultures increased by between 17- and 22-fold, while the number of MSCs multiplied by only 5- to 6-times (Figure 1C). These results indicate that a subset of drugs can kill both cancer cells and MSCs despite the huge differences in the proliferation rate.

**Figure 2:**
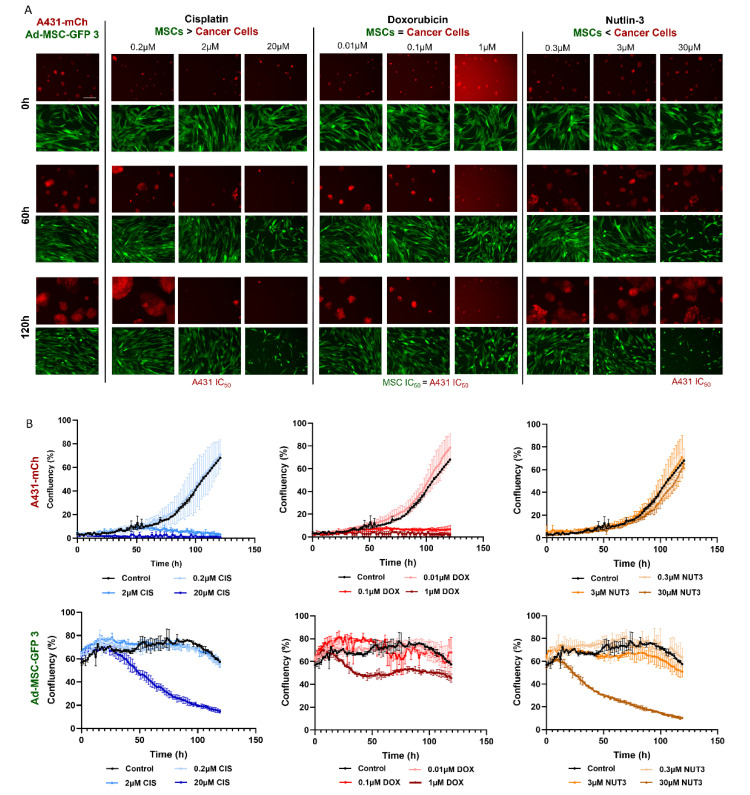
Live cell images of Ad-MSC-GFP 3 and A431-mCh cells after 120 h of treatment with cisplatin, doxorubicin, or nutlin-3. (**A**) Images were taken every 105 min for 120 h, and representative images were selected at 0, 60, and 120 h to show the progress of cultures under treatment with the indicated drugs and concentrations. Note that at 1 µM of doxorubicin background fluorescence significantly increased due to the drug’s autofluorescence. (**B**) The changes in confluency (%) were calculated from 3 independent experiments using image analysis. Due to the larger size of the MSCs, confluency of MSCs is higher than that of cancer cell cultures from the start of the experiment. Upper row left from right: A431-mCherry cells treated with different concentrations of cisplatin, doxorubicin, or nutlin-3. Bottom row left from right: Ad-MSC-GFP 3 cells treated with different concentrations of cisplatin, doxorubicin, or nutlin-3.

A 120-h live cell imaging was also conducted to visually confirm the effects of compounds on different cell types using mCherry expressing A431-mCh cancer cells and Ad-MSC-GFP 3 MSC cultures (Figure 2). Three drugs were selected for the experiments based on the selective toxicity either to cancer cells (cisplatin), to MSCs (nutlin-3), or showing similar efficiency against both (doxorubicin). As the aim of chemotherapy is to kill cancer cells, we used concentrations equal to A431-mCh’s IC_50_ values with two additional, 10-fold lower and higher doses. A431-mCh cells responded to drugs as expected, while the lower concentrations of cisplatin and doxorubicin did not alter growth kinetics compared to untreated controls, both IC_50_ and higher concentrations of the drugs significantly reduced the number of viable colonies. Since nutlin-3 was engineered to target only p53 wild-type cells, the drug had no effect on p53 mutant A431-mCh cultures in either concentration. Interestingly, despite cytotoxicity results suggesting that MSCs and cancer cells share remarkably similar sensitivity to doxorubicin, no significant decrease was observed in Ad-MSC-GFP 3 cultures after 120 h of treatment. Significant drops in confluency were only seen at concentrations of 2-fold of cisplatin and 3-fold of nutlin-3 IC_50_ values found on Ad-MSC-GFP 3. This outcome suggests that MSCs, rather than dying off after treatment, choose a different path, such as cell cycle arrest, senescence, or differentiation, and can only be killed with significantly higher concentrations than the IC_50_ observed in a cytotoxicity assay.

### 3.2. MSCs Suffer Less Treatment Induced DNA-Damage despite Having Similar Sensitivity to Chemotherapeutics

**Figure 3:**
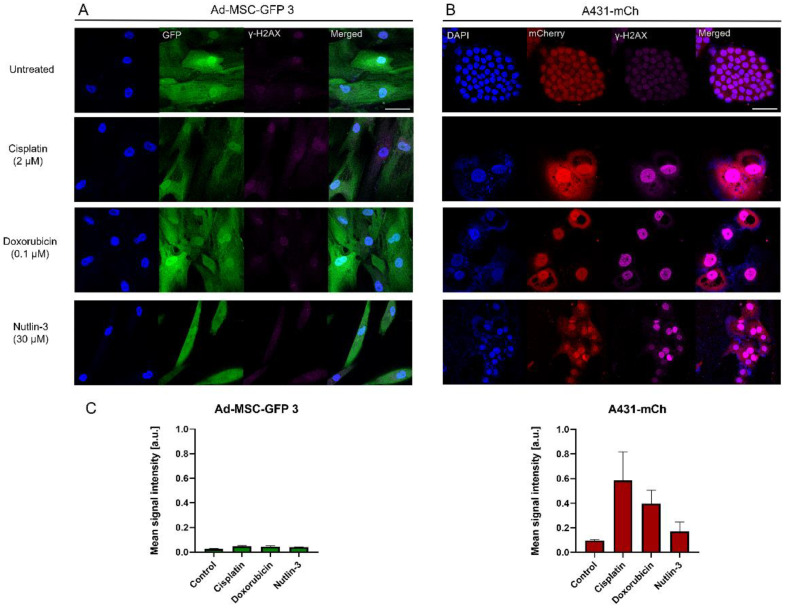
Analysis of DNA double-stranded breaks in (**A**) Ad-MSC-GFP 3 and (**B**) A431-mCh cell lines at 120 h, treated with IC_50_ concentrations of A431 cells with γ-H2AX intensity quantification (**C**). Scale bar on the confocal images is 50 µM.

As it seems, MSCs can also be affected by chemotherapeutics despite possessing a significantly lower proliferation rate compared to cancer cells, but intriguingly avoiding cell death in some way. To further examine the response of cancer cells and MSCs to drugs, DNA damage was investigated. DNA double-stranded breaks in the nuclei of treated cells were detected using phosphorylated γ-H2AX staining. Cisplatin, doxorubicin, and nutlin-3 can directly or indirectly cause DNA-damage in cells, therefore, we investigated DNA-damage in the same two cell lines as above and optimized the treatment conditions according to A431-mCh cells’ IC_50_ values. Important differences were found in the two cell lines (Figure 3A,B). All 3 drugs caused massive DNA damage in A431-mCh cells (Figure 3B), even nutlin-3, which, according to the live cell imaging experiments, did not reduce cell number after 120 h of treatment. In contrast, no DNA break was detected in Ad-MSC-GFP 3 cells, not even after nutlin-3 treatment, despite both cytotoxicity and video microscopy experiments showing significantly decreased viability at 3-fold IC_50_ concentrations (30 µM). Increasing cisplatin concentration to 10 µM (IC_50_ concentration of Ad-MSC-GFP3 cells) significantly increased the number of double-stranded breaks in MSCs, while almost completely eradicating A431 cells (Supplementary Figure S3A).

### 3.3. MSCs Show No Different Chemotherapeutics Related ROS Induction Compared to Cancer Cells

**Figure 4:**
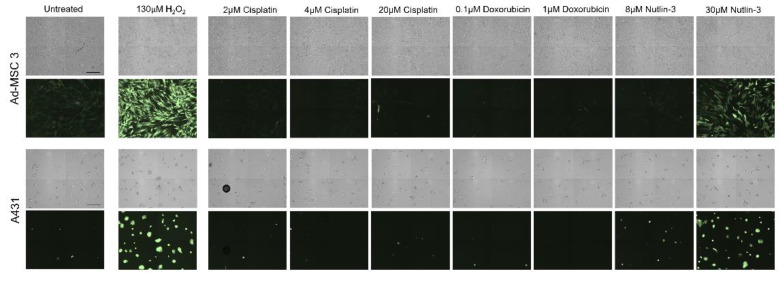
Assessing ROS content after drug treatment in Ad-MSC 3 and A431 cell lines. Brightfield (**upper** row) and fluorescent (**lower** row) images of A431 and Ad-MSC 3 after 1 h of drug exposure to indicated concentrations. Scale bar is 300 µM.

Not only is the increased metabolic rate able to induce large amounts of cytotoxic ROS in cancer cells, but several anticancer agents can also generate ROS via the electron transport chain. This induced oxidative stress can damage the cells and lead to ROS-mediated cell death in cancer cells and MSCs. As differences in DNA damage cannot explain the contrast in drug response between cancer cells and MSCs, reactive oxygen species (ROS)-related cell death mechanisms were also examined. Cells were treated with cisplatin, doxorubicin, and nutlin-3 at different concentrations for 1 h to elucidate ROS induction after treatment (Figure 4). Cisplatin and doxorubicin did not induce ROS, even when cells were treated with higher concentrations. Strikingly, 30 µM of nutlin-3 generated a considerable amount of ROS in both Ad-MSC 3 and A431 cells, resulting in an almost similar increase as 130 µM of the H_2_O_2_ control treatment. According to live cell imaging, 30 µM of nutlin-3 was more toxic to Ad-MSC-GFP 3 than A431, suggesting that cancer cells can possibly manage ROS-related damage more efficiently.

### 3.4. MSCs Avoid Apoptosis after Drug Treatment

**Figure 5:**
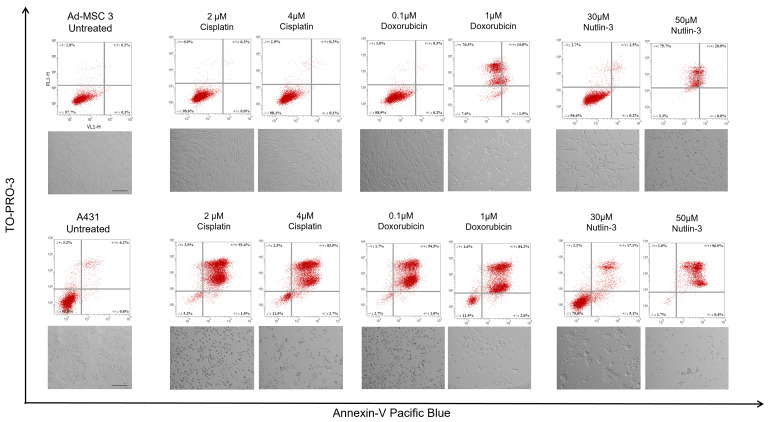
Apoptosis was induced only in A431 cancer cells. 2 µM and 4 µM cisplatin, 0.1 µM and 1 µM doxorubicin, and 50 µM nutlin-3 treatment caused apoptosis in A431cells (**lower** row). 30 µM nutlin-3 treatment shows low number of apoptotic A431 cells. None of these concentrations induced apoptosis in Ad-MSC 3 cells (**upper** row).

The main goal of chemotherapy is to induce apoptosis in cancer cells. Therefore, apoptosis induction was investigated after 120 h of treatment with cisplatin, doxorubicin, or nutlin-3. Cells were stained with Annexin-V-Pacific Blue and TO-PRO-3, and the ratio of Annexin-V positive cells was measured by flow cytometry (Figure 5). A431 cells showed apoptosis after all treatment with only a modest apoptotic response to 30 µM nutlin-3. Strikingly, apoptosis was not observed in MSCs during our experiments even when the drug concentrations were increased sometimes by 10-fold. Previously, it was shown that the IC_50_ value of doxorubicin is 0.1 µM in both cell lines, however, while this concentration caused apoptosis in almost all A431 cells, apoptosis was not detected in Ad-MSC 3 cultures. Moreover, when doxorubicin concentration was increased 10-fold, only late necrosis was found. Similarly, treatment with 30 µM of nutlin-3, which is 300% of the Ad-MSC 3 IC_50_ value, resulted in only a slight increase in the number of apoptotic MSCs. Cisplatin caused virtually no apoptosis compared to untreated controls.

### 3.5. Neither MSCs nor Cancer Cells Become Senescent after Drug Treatment

**Figure 6:**
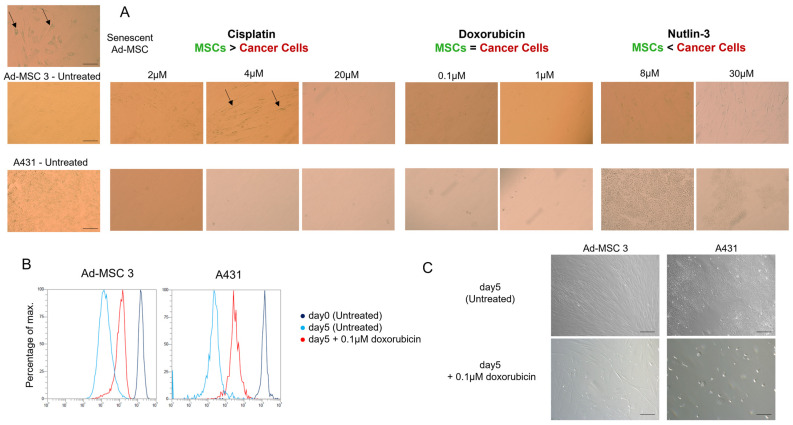
Senescence-associated β-galactosidase (SA-β-gal) staining in A431 and Ad-MSC 3 cells. (**A**) SA-β-gal shows slight positivity to 4 µM cisplatin treatment, and positive control (Senescent Ad-MSC 3). (**B**) 0.1 µM doxorubicin five-day treatment decreased the fluorescent signal in CytoTell assay in both cell types. (**C**) Cells were imaged under light microscope at 10× objective magnification.

Senescence can be an induced cell response to chemotherapy treatment. Cellular senescence is a type of cell death, where senescence-associated mechanisms arrest cell growth. The senescence-associated β-galactosidase (SA-β-gal) staining kit detects the increased β-galactosidase enzyme activity in cells. SA-ß-Gal catalyzes the hydrolysis of X-gal dye, which produces a blue color in senescent cells. The positive control cells (senescent Ad-MSC 3) at high passage number (p27) and after 4 µM cisplatin treatment show a slightly increased SA-ß-Gal staining in MSCs, but not in cancer cells (Figure 6A). On the other hand, doxorubicin treatment decreased proliferation in both cell lines (Figure 6B), reduced cell number in the MSC population, and killed most of the cancer cells (Figure 6C).

## 4. Discussion

Although MSCs are one of the most abundant cell types in the TME, their response to the applied chemotherapies is mostly unknown and a systematic drug screen on MSCs has never been performed before [11]. In this work, we compared the drug sensitivity of 6 MSC lines isolated from cancer-free individuals and 3 widely used cancer cell lines to 9 structurally and mechanistically different compounds and characterized their response to 3 of these in detail.

In our screening, MSCs showed strong resistance, sometimes 10,000-fold, to DNA intercalation (cisplatin), topoisomerase I and II inhibition (irinotecan, mitoxantrone), and microtubule disturbances (vinblastine). Similar results were observed previously by others testing the same compounds on MSCs. Nicolay et al. showed bone marrow (BM)-derived MSCs and differentiated fibroblasts exhibit resistance to irinotecan and etoposide, most likely due to efficient DNA repair [12]. Both Mueller and Liang et al. found that BM- and adipose tissue-derived MSCs pose resistance to cisplatin, vincristine, and camptothecin, and rapidly recover after drug exposure [13,14]. It is common knowledge that chemotherapy targets rapidly dividing cancer cells, therefore, healthy tissues are affected significantly less than tumors. The widely accepted explanation is that quiescent and slow cycling normal cells have less active DNA synthesis and other proliferation-related activities, which are usually targeted by chemotherapeutics [15,16]. Considering MSCs’ significantly lower proliferation rate, the observed resistance can be explained.

Despite MSCs being considered as chemotherapy-resistant in the related literature, our experiments brought striking results that challenge this view. Four chemotherapeutic compounds proved to be surprisingly effective against all cell lines with MSCs having no notable advantage over cancer cells. Intrastrand crosslinking (bendamustine), topoisomerase II inhibition (doxorubicin), ROS-damage (TPEN), and antimetabolite-induced DNA damage (methotrexate) affected MSCs similarly to cancer cells. No direct testing of bendamustine on MSCs was reported previously, however, available results are conflicting and suggest that using the drug can have a negative effect on stem cell mobilization in patients; however, simultaneously, low toxicity was observed on stem cell cultures in vitro [17,18]. Doxorubicin and mitoxantrone were found to be slightly toxic to MSCs in some studies, but the main differences were primarily in the expression of a few certain genes (e.g., connexin 43, alkaline phosphatase, troponin T) rather than complete phenotypic changes or cell death. MSCs isolated from doxorubicin-treated rats showed activated DNA damage response pathway, S-phase arrest, and increased sub-G1 subpopulation, while mitoxantrone caused premature senescence in dental pulp MSCs and dermal fibroblasts [19,20]. Importantly, in these works, sensitivity of MSCs was not compared to cancer cells. TPEN is a cell permeable Zn^2+^ chelator used to induce oxidative stress through the dysregulation of ROS detoxification [21,22]. MSCs’ sensitivity to ROS is still unclear: some reports suggest that MSCs have low base levels of ROS with a high amount of the antioxidant glutathione, which protects the cells during oxidative attacks [23]. In contrast, others showed MSCs are more susceptible to ROS-induced damage than differentiated cells and respond to ROS-inducing agents with DNA damage and senescence; moreover, exogenous antioxidants were required to restore oxidative stress resistance [24,25]. In our experiments, TPEN treatment was equally toxic to both MSCs and cancer cells, suggesting MSCs have no advantage against ROS-mediated damage. The antimetabolite methotrexate, used to treat cancer and rheumatoid arthritis, interferes with thymidine synthesis through inhibiting dihydrofolate reductase (DHFR). Methotrexate’s effects rely extensively on DHFR expression and structure, mutations in the active site of the enzyme, or lower expression levels render cells resistant to the drug [26]. In accordance with our findings, MSCs considered relatively resistant to antimetabolites, such as 5-fluorouracil and gemcitabine, keep their cellular characteristics despite treatment probably as a result of increased DNA metabolism and multidrug resistance transporter expression [27]. The only significant outlier was the A431 cell line, which proved to be hypersensitive to the compound, which could be the result of the cell-specific DHFR status or the fact that A431 is the only p53-mutant in the cell line panel [28]. Nutlin-3, an effective MDM2-p53 interaction inhibitor, was designed to selectively kill wild-type p53 expressing cells [29]. In our assay, only A431 proved to be resistant to nutlin-3, the only cell line with mutated p53 expression.

Finding similar drug sensitivities in both MSCs and cancer cells is even more surprising if we consider that, despite the comparable responses seen in the cytotoxicity assays, the two cell types react quite differently to the same toxic insults. Doxorubicin was equally toxic to MSCs and cancer cells in the cytotoxicity assays, however, in MSCs there were less DNA double-stranded breaks (DSBs), suggesting an increased DNA repair, and while MSCs are known to successfully repair DSBs after treatment with drugs such as bleomycin [30], it still was not enough to protect cells from the cytotoxic effects of doxorubicin. Furthermore, nutlin-3 caused DNA damage only in cancer cells, yet only killed p53-wt expressing MSCs, which indicates that the amount of DNA DSBs cannot properly predict survival after drug treatment.

Treatment-induced ROS generation was also assessed, but, surprisingly, ROS levels increased significantly only by nutlin-3 in both cancer cells and MSCs. While others hypothesized that nutlin-3 induces ROS through increasing p53 and mitochondrial translocation levels, which in turn result in elevated ROS [31], in our assay, both p53-wt (MSC) and -mutant (A431) cells showed a robust increase, but, again, only MSCs were killed off after treatment.

The most striking result was, unquestionably, the inability of MSCs to die by apoptosis. There are some findings by others suggesting MSCs have an elevated apoptotic threshold [13], but the extent of this phenomenon was never shown before. Some annexin-positive, late apoptotic cells were detected in MSC cultures after treatment with high concentrations of doxorubicin and nutlin-3 (1 µM and 50 µM, respectively). However, no distinct apoptotic populations were found, showing that treatments causing massive apoptosis in cancer cells only modestly activate the apoptotic pathways in MSCs. Nevertheless, the same high concentrations of doxorubicin and nutlin-3 caused necrotic cell death in MSCs, suggesting that mesenchymal stem cells can be killed with chemotherapy, but with significantly higher doses and activating different cell death mechanisms. Surprisingly, when the cisplatin concentration was raised to the IC50 level of Ad-MSC-GFP 3 cells (10 µM), notable amounts of late apoptosic cells were observed, suggesting that apoptosis in mesenchymal stem cells can be induced with specific DNA damage insults. We were not able to induce apoptosis in MSCs using several concentrations of mitoxantrone, staurosporine (multikinase inhibitor), H_2_O_2_ (oxidative stress), and cobalt chloride (hypoxia inducer) in our experiments (data not shown). Other groups reported possible chemotherapy-induced apoptosis in MSCs, however, these works mostly show only a slight increase in cleaved caspase-3 or annexin-V as a marker of apoptotic cells [12,20,27,32]. This observation could mean MSCs are inherently resistant to apoptosis or more preferably choose other pathways than apoptosis.

To test whether MSCs switch to senescence rather than cell death, the function of senescence-associated ß-galactosidase (SA-ß-GAL) was investigated 12 days after drug treatment, but no significant differences were found. The only alteration found was the decrease in proliferation rate in both cell types. Due to cancer cells being proven to be dying after drug treatment, one possible explanation could be that MSCs slow down their cell cycle progression and buy some time to avoid apoptosis and follow another pathway.

In summary, our results point out important differences in the drug response of MSCs and cancer cells, showing how complex the role of TME could truly be during chemotherapy. These observations could help rational drug design and the development of drug combinations targeting both MSCs and cancer cells in the TME.

## Data Availability

Not aapplicable.

## Supplementary Materials

The following supporting information can be downloaded at: https://www.mdpi.com/article/10.3390/cells11192942/s1. Additional methods section: 1. Establishment of a novel BM-MSC cell line; 2. Characterization of BM-MSC cell line; 3. Comparison of transfected and non-transfected cell lines viability. Supplementary Figure S1: Characterization BM-MSC. Supplementary Figure S2: Comparison of drug sensitivity of GFP or mCherry expressing and parental cell lines treated with cisplatin, doxorubicin, or nutlin-3. Supplementary Table S1. Mean IC50 values ± SD of 9 drugs measured across 9 cell lines. Supplementary Figure S3: Effects of 10 µM cisplatin on double-strand break and apoptosis induction in Ad-MSC 3 and A431 cells.
