# Supplementary material for: Comparison of Different Clinical Chemotherapeutical Agents’ Toxicity and Cell Response on Mesenchymal Stem Cells and Cancer Cells
Source: Cells. 2022 Sep 20;11(19):2942. doi: 10.3390/cells11192942 (PMC9563435; doi:10.3390/cells11192942)
Supplement: Supplementary file 1 [file cells-11-02942-s001.zip › cells-1830480-supplementary.pdf]

Supplementary Material to Comparison of different clinical chemotherapeutical agents' toxicity and cell response on mesenchymal stem cells and cancer cells by Vajda et al.

Establishment of a novel BM-MSC cell line

BM-MSC cell line was isolated from a 4-year-old donor during an orthopedic surgery and characterized according to International Society for Cellular Therapy's (ISCT) guidelines (32). All procedures were approved by the Hungarian Medical Research Council (license number: 24083-3/2013/HER) and the study was performed accordance with the Declaration of Helsinki, the collection and the scientific use of tissue samples was carried out according to the written and oral information shared with the participants and their parents. A written permission from the donor's parents was also given. The BM-MSCs were characterized according to ISCT guidelines for mesenchymal stem cell identification. The plastic-adherence, expression of specific cell surface CD-markers (positivity to CD44, CD73, CD90 and CD105 and negativity to CD14, CD34, CD45, CD117, CD133 and HLA-DR) and the ability to differentiate to adipogenic, osteogenic and chondrogenic lineages under specific conditions were investigated. CD protein recognizing antibodies were purchased from BD Pharmingen (CD44-FITC, CD73-PE, CD90-APC, CD105-PE, CD117-PE, HLA-DR-PE, CD45-PE), Beckham Coulter (CD14-FITC), Beckton Dickinson (Franklin Lakes, New Jersey, United States) (CD34-FITC) and Miltenyi Biotec (Surrey, United Kingdom) (CD133-APC) and the expression profile of the BM-MSCs was measured using an Attune™ Nxt flow cytometer (Thermo Fisher Scientific). To assess the differentiation potential of different MSCs, cells were differentiated into adipocyte, osteoblast, and chondrocyte as described previously (Szepesi et al. (33) namely StemPro® osteogenic (A10072-01, Thermo Fisher Scientific), adipogenic (A10070-01, Thermo Fisher Scientific) and chondrogenic (A10071-01, Thermo Fisher Scientific) induction media were used to induce differentiation of BM-MSCs and the treatment was continuously maintained for 14 days. To assess successful differentiation the cells were fixed using 4% PFA on day 14 and stained with 2% Alizarin Red S for calcium crystals (osteogenic), with 0.5% Oil Red O dye for intracellular lipid droplets (adipogenic) and with 0.5% 1,9-dimethyl-methylene-blue for glycosaminoglycans (chondrogenic). Cells were cultured in DMEM-F12 media (see above), renewed in every 72 hours, and passaged at 80% confluency.

Characterization of human Bone-Marrow Mesenchymal Stem Cells

For our experiments we used a panel of MSC/fibroblast cell lines consisting of three adipose tissue-derived MSCs obtained from healthy donors (Ad-MSC 1, Ad-MSC 2, Ad-MSC 3) and one immortalized subline of Ad-MSC 1 (iAd-MSC 1) which were previously characterized (34), with one additional, commercially available fibroblast cell line (HFF). While, according to the currently dominant theories, TME MSCs mostly originating from local adipose tissues, some reports suggesting that bone marrow could be another source for intratumoral MSC recruitment (35). To address this possibility, here we describe a novel bone marrow-derived MSC line, the BM-MSC. BM-MSCs were proved to be plastic adherent and showed spindle-like morphology. Expression of CD44, CD73, CD90, CD105 and lack of CD34, CD45, CD117 and CD133 was detected. HLA-DR were observed nearly 50% of the BM-MSCs.

(Supplementary Figure S1A). BM-MSCs were in fact multipotent stem cells and were able to differentiate into adipogenic, osteogenic and chondrogenic lineages under specific conditions. Alizarin Red S binds to calcium deposits, showing strong osteogenic differentiation, Oil Red O dye stains the intracellular lipid vesicles containing triglycerides and diacylglycerols proving successful adipogenic differentiation, while in chondrocytes 1-9-dimethyl-methylen blue labelled glycosaminoglycans like chondroitin sulfate, keratin sulfate and heparan sulfate of the extracellular matrix produced by chondrocytes (Supplementary Figure S1B). Interestingly, the differentiation potential of the BM-MSCs showed strong preference to osteogenic and chondrogenic lineages as adipogenic differentiation was much weaker compared to the other two (Supplementary Figure S1B).

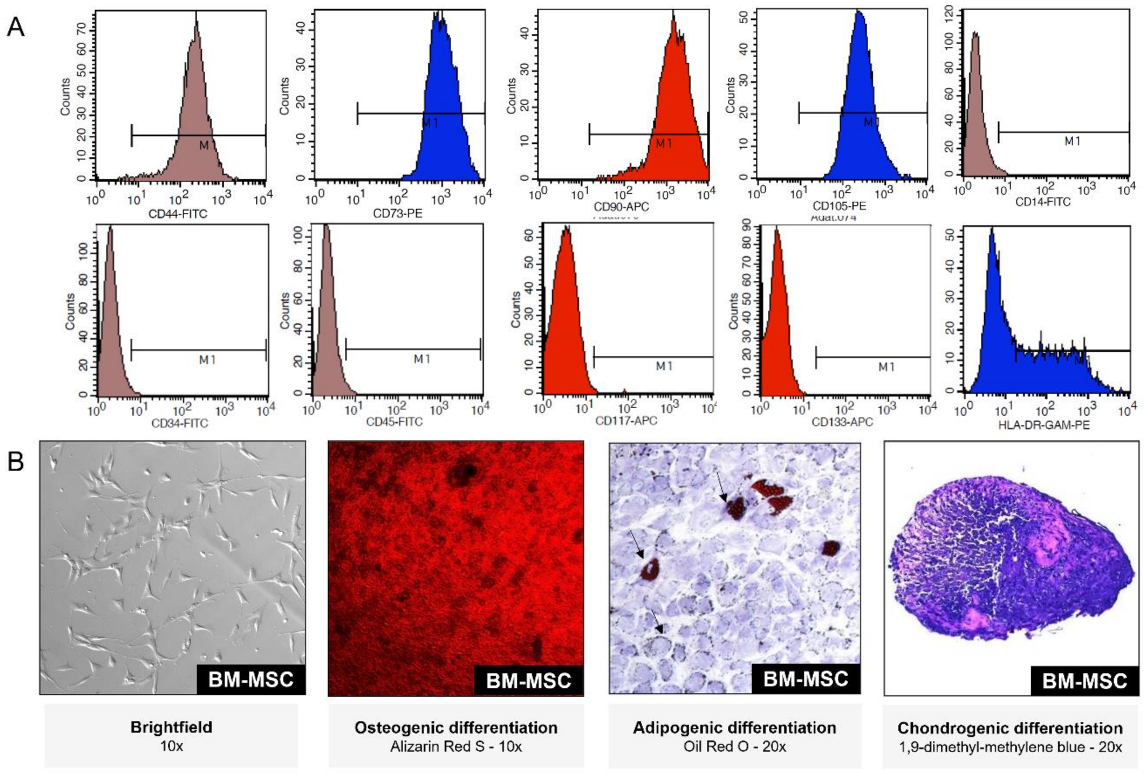

Supplementary Figure S1. Characterization of BM-MSC. (A) Flow cytometry analysis of MSC cell surface markers. Expression of CD44, CD73, CD90, CD105 and the lack of CD14, CD34, CD45, CD117, CD133 and HLA-DR endothelial and HSC markers were detected. (B) Investigation of the differentiation potential of BM-MSC cells. Alizarin Red S stains the osteocytes, Oil Red O the lipid vesicles in adipocytes and 1,9-dimethylmethylene glycosaminoglycans in the chondrocytes.

Comparison of transfected and non-transfected cell lines viability

Fluorescent protein expressing cells can be used for long-term live cell imaging and for monitoring viability in various environments. To demonstrate that the lentiviral transduced cells did not change during gene delivery procedure, dose-response curves of GFP/mCherry expressing and parental cells were compared using three different chemotherapeutic agents: cisplatin, doxorubicin, and nutlin-3 (Supplementary Figure S2A and S2B). Average IC_{50} values are calculated from three independent experiments and are shown in Supplementary Figure S2C. The results demonstrated no significant differences between the fluorescent protein

expressing and parental cell lines, indicating that the genetic modification did not alter drug sensitivity and therefore transduced cells can be used for further analysis.

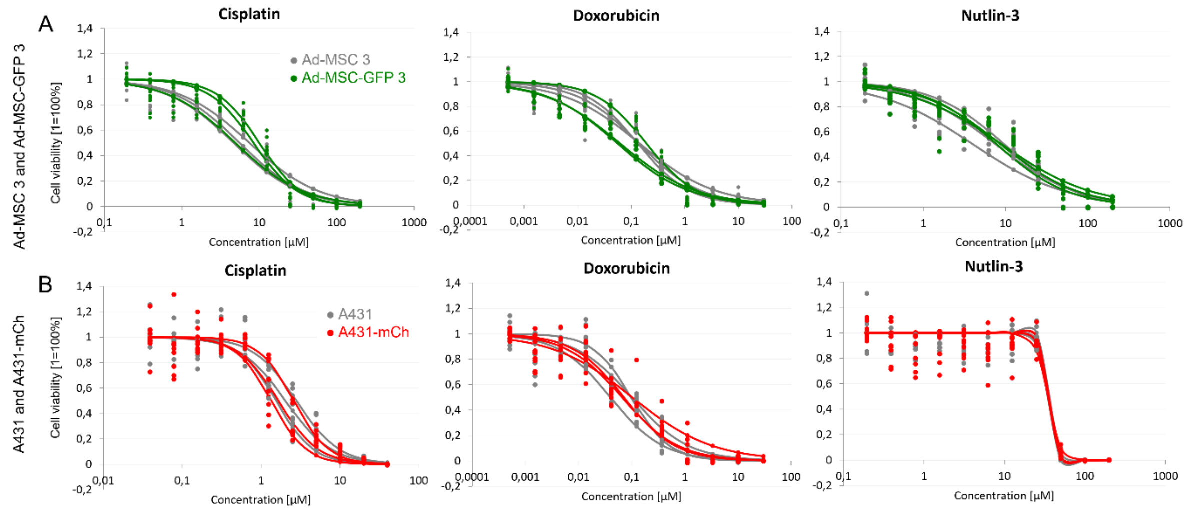

C	Cisplatin			Doxorubicin			Nutlin-3		
	**IC_{50} [µM]**	log_{10} IC_{50}	st.dev. log_{10} IC_{50}	**IC_{50} [µM]**	log_{10} IC_{50}	st.dev. log_{10} IC_{50}	**IC_{50} [µM]**	log_{10} IC_{50}	st.dev. log_{10} IC_{50}
**Ad-MSC 3**	**7,4445**	0,871	0,161	**0,1369**	-0,863	0,0146	**6,6569**	0,823	0,197
**Ad-MSC-GFP 3**	**5,7818**	0,762	0,103	**0,0862**	-1,06	0,298	**8,4827**	0,928	0,0306
**A431**	**2,0526**	0,312	0,128	**0,0729**	-1,13	0,228	**33,802**	1,52	0,0905
**A431-mCh**	**1,7787**	0,25	1,47	**0,0780**	-1,1	0,102	**33,885**	1,53	0,0153

Supplementary Figure S2. Comparison of drug sensitivity of GFP or mCherry expressing and parental cell lines treated with cisplatin, doxorubicin, or nutlin-3. (A) Dose response curves of Ad-MSC 3 (grey) and Ad-MSC-GFP 3 (green) cells. (B) Dose response curves of A431 (grey) and A431-mCh (red) cells. (C) IC_{50} values, calculated $log_{10}IC_{50}$ and standard deviation (st.dev.) are represented in the table.

[µM]	Ad-MSC-GFP 1	iAd-MSC-GFP 1	Ad-MSC-GFP 2	Ad-MSC-GFP 3	HFF	BM-MSC	MCF7-GFP	MESSA-mCherry	A431-mCherry
**Cisplatin**	9,63 ± 4,47	16,64 ± 6,6	6,45 ± 1,86	10,58 ± 1,11	11,71 ± 3,15	3,38 ± 2,81	1,15 ± 0,54	0,69 ± 0,14	2,09 ± 0,47
**Irinotecan**	10,54 ± 11,33	24,21 ± 21,4	7,03 ± 1,65	10,2 ± 19,17	16,36 ± 6,73	13,77 ± 23,93	1,77 ± 1,44	4,26 ± 2,05	1,1 ± 1,14
**Vinblastine**	4,82 ± 2,79	1,19 ± 0,34	19,54 ± 24,32	0,13 ± 0,01	2,38 ± 7,14	0,14 ± 23,69	0,0018 ± 0,003	0,00092 ± 0,00229	0,00367 ± 0,01409
**Bendamustine**	428,86 ± 173,13	556,23 ± 13,01	146,76 ± 30,07	126,95 ± 29,59	200,54 ± 158,19	301,38 ± 62,23	193,16 ± 22	308,73 ± 95,29	341,54 ± 224,12
**Doxorubicin**	0,80 ± 0,22	0,18 ± 0,074	0,11 ± 0,05	0,22 ± 0,04	0,34 ± 0,25	0,06 ± 0,02	0,21 ± 0,1	0,16 ± 0,03	0,11 ± 0,05
**Mitoxantron**	0,91 ± 0,38	0,3 ± 0,2	0,30 ± 0,17	0,29 ± 0,15	0,62 ± 0,27	0,4 ± 0,4	0,06 ± 0,01	0,02 ± 0,01	0,18 ± 0,14
**Methotrexate**	500 ± 0 (nt)	475,48 ± 40,41	355,72 ± 17,35	500 ± 0	444,92 ± 48,88	500 ± 0	500 ± 0	500 ± 0	0,09 ± 0,02
**TPEN**	3,53 ± 0,18	2,81 ± 1,01	3,85 ± 1,46	3,68 ± 0,08	4,45 ± 0,82	3,69 ± 0,35	3,93 ± 1,59	2,46 ± 1,12	1,51 ± 0,97
**Nutlin-3**	4,96 ± 0,84	6,59 ± 2,5	2,82 ± 0,8	8,48 ± 0,59	5,46 ± 2,3	4,01 ± 4,52	1,5 ± 0,24	3,09 ± 0,46	24,48 ± 1,93

Supplementary Table S1. Mean IC50 values ± SD (µM) of 9 drugs measured across 9 cell lines. Note: Methotrexate was only toxic to A431-mCherry cells.

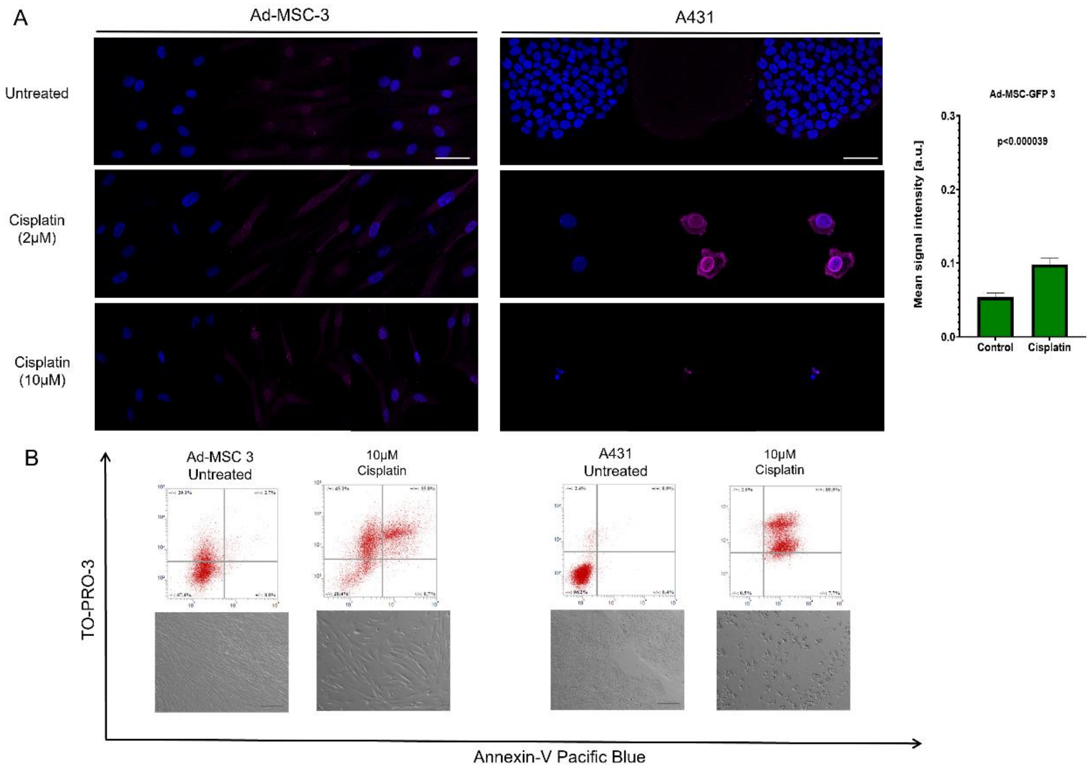

Supplementary Figure S3. Effects of 10 μM cisplatin on double-strand break and apoptosis induction in Ad-MSC 3 and A431 cells. (A) Fluorescent images showing γ-H2AX-positive double-strand DNA breaks in A431 and Ad-MSC 3 cells after 5 days of 10 μM cisplatin treatment. Nuclei were stained with DAPI (blue); DNA double-strand breaks were detected using γ-H2AX (magenta) staining. Scale bar on the confocal images is 50μm. The bar graph shows the quantification of the γ-H2AX signal in control and 10 μM cisplatin treated Ad-MSC 3 cells. (B) Apoptosis analysis of Ad-MSC 3 and A431 cells treated with 10 μM cisplatin for 5 days.
